# Changing educational inequalities in sporting inactivity among adults in Germany: a trend study from 2003 to 2012

**DOI:** 10.1186/s12889-017-4478-2

**Published:** 2017-06-06

**Authors:** Jens Hoebel, Jonas D. Finger, Benjamin Kuntz, Lars E. Kroll, Kristin Manz, Cornelia Lange, Thomas Lampert

**Affiliations:** 1 Unit of Social Determinants of Health, Department of Epidemiology and Health Monitoring, Robert Koch Institute, General-Pape-Straße 62-66, 12101 Berlin, Germany; 2 Unit of Health Behaviour, Department of Epidemiology and Health Monitoring, Robert Koch Institute, General-Pape-Straße 62-66, 12101 Berlin, Germany

**Keywords:** Social determinants of health, Physical inactivity, Exercise, Sports, Physical activity, Sports participation, Health inequalities, Socioeconomic inequalities in health

## Abstract

**Background:**

Social inequalities in health can be explained in part by the social patterning of leisure-time physical activity, such as non-participation in sports. This study is the first to explore whether absolute and relative educational inequalities in sporting inactivity among adults have changed in Germany since the early 2000s.

**Methods:**

Data from four cross-sectional national health surveys conducted in 2003 (*n* = 6890), 2009 (*n* = 16,418), 2010 (*n* = 17,145) and 2012 (*n* = 13,744) were analysed. The study population was aged 25–69 years in each survey. Sporting inactivity was defined as no sports participation during the preceding 3 months. The regression-based Slope Index of Inequality (SII) and Relative Index of Inequality (RII) were calculated to estimate the extent of absolute and relative educational inequalities in sporting inactivity, respectively.

**Results:**

Sporting inactivity was consistently more prevalent in less-educated groups. The overall prevalence of sporting inactivity declined significantly over time. However, the decline was observed only in the high and medium education groups, while no change was observed in the low education group. Both absolute and relative educational inequalities in sporting inactivity were found to have widened significantly between 2003 (SII = 0.30, 95% CI = 0.25–0.35; RII = 2.08, 95% CI = 1.83–2.38) and 2012 (SII = 0.41, 95% CI = 0.37–0.45; RII = 3.44, 95% CI = 3.03–3.91). Interaction analysis showed that these increases in inequalities were larger in the younger population under the age of 50 than among the elderly.

**Conclusions:**

The findings suggest that the gap in sports participation between adults with high and low educational attainment has widened in both absolute and relative terms because of an increase in sports participation among the better educated. Health-enhancing physical activity interventions specifically targeted to less-educated younger adults are needed to prevent future increases in social inequalities in health.

**Electronic supplementary material:**

The online version of this article (doi:10.1186/s12889-017-4478-2) contains supplementary material, which is available to authorized users.

## Background

Sports activities often involve high-intensity endurance-oriented physical exercise, which is more strongly associated with health benefits than other types of physical activity [1, 2]. Consequently, sports promotion at the population level is considered an effective strategy for enhancing public health and, accordingly, sports promotion policies have been implemented in many European countries during recent years [3]. The German government, for example, initiated a National Action Plan in 2008 to promote physically active lifestyles among the general population of Germany [4].

In Germany and most other European countries, a higher level of leisure-time physical activity including sports activity is associated with higher social position among adults [5–8]. A systematic review demonstrated that among different indicators of social position, education produces the most stable relationships with physical activity habits [9]. Higher education can promote sports participation through several individual and social factors, such as self-efficacy, outcome expectancies, financial resources, social networks, and environmental conditions such as neighbourhood safety [10]. Educational differences in physical activities during leisure time, such as sports activity, have been found to partly explain social inequalities in health outcomes [11]. Therefore, narrowing the educational gap in health-enhancing physical inactivity has the potential to reduce social inequalities in health.

When examining whether and how the magnitude of social inequalities in health risks in a population changes over time, there can be a substantial difference depending on whether absolute or relative inequalities are taken into account [12–14]. While absolute inequalities are typically expressed by rate differences between social groups, relative inequalities are quantified in rate ratios. Research suggests that a reduction of absolute inequalities occurs under a wider range of conditions than a reduction of relative inequalities [13]. Consequently, evidence shows that in the case of declining rates overall, relative inequalities can increase, while absolute inequalities do not [15]. It is therefore recommended that inequalities in both absolute and relative terms are considered when monitoring trends over time [16, 17].

While research indicates an increase in the overall prevalence of sports activity in Germany [18], no study to date has examined whether social inequalities in sports activity among adults have changed in Germany since the beginning of the 2000s. Against the background of increasing overall sports participation and intensified physical activity promotion in Germany, this study aims to explore changes over time in the magnitude of absolute and relative educational inequalities in sporting inactivity in the German adult population between 2003 and 2012.

## Methods

### Study design and data collection

The study was based on data from four repeated national telephone health surveys of the adult population in Germany. The surveys are part of a nationwide Health Monitoring System administered by the Robert Koch Institute (RKI) on behalf of the German Federal Ministry of Health [19, 20]. The aim of the regularly conducted cross-sectional surveys is to provide current data on population health, health determinants, and the use of health services for national and European health reporting systems, health policies, and public health research.

The first German-wide telephone health survey was conducted in 2003 and continued by the ‘German Health Update’ (GEDA) surveys in 2009, 2010, and 2012 [20]. For the present study, we used the data from all these four cross-sectional surveys. Each of the surveys was based on a two-stage sampling procedure. First, random samples of telephone numbers from the entire German fixed-line network were generated using random digit dialling. Second, one adult member of each contacted household was randomly selected for interview. Sample sizes and characteristics of the study populations are shown in Table 1. Data were collected by computer-assisted telephone interviewing; interviews took on average approximately half an hour and included standardised questions about health status, health behaviours, healthcare utilization and sociodemographics. Further information on survey design, contents, and response rates can be found elsewhere [20, 21].

**Table 1:** Characteristics of the study population by survey year

	2003	2009	2010	2012
	(*n* = 6890)	(*n* = 16,418)	(*n* = 17,145)	(*n* = 13,744)
Sex, % (n)				
Men	50.3 (3224)	50.2 (6930)	50.2 (7330)	50.3 (6799)
Women	49.7 (3666)	49.8 (9488)	49.8 (9815)	49.7 (6945)
Age, years				
Mean ± SD	46.2 ± 12.4	46.8 ± 12.4	46.8 ± 12.3	46.9 ± 12.1
Age group, % (n)				
25–39 years	35.4 (2237)	31.3 (4761)	30.7 (5015)	30.0 (3162)
40–49 years	24.9 (2073)	27.6 (4924)	27.9 (4992)	27.5 (3665)
50–59 years	19.4 (1363)	22.0 (3704)	22.6 (3931)	24.4 (3592)
60–69 years	20.3 (1217)	19.1 (3029)	18.8 (3207)	18.2 (3325)
Education, % (n)				
Low	37.5 (2003)	33.5 (3580)	33.1 (3656)	28.6 (2399)
Medium	47.0 (3249)	50.1 (8110)	49.1 (8212)	51.3 (6847)
High	15.6 (1605)	16.4 (4698)	17.8 (5254)	20.1 (4476)

%, weighted percentage (extrapolated to the population of Germany)

*n*, unweighted number of cases in the sample

*SD*, standard deviation

### Measures

Sports participation was determined by asking respondents, “Think about the last 3 months. Have you done any sport?” (“Yes”/“No”). The term “sport” was not specified in the question wording; however, in German, the term “sport” has a broad meaning and is generally thought to include not only competitive club sports but also physical exercise to improve or maintain physical fitness. In our analysis, “sporting inactivity” was defined as no sports participation during the preceding 3 months.

To measure educational attainment, respondents were asked for their highest level of school achievement and their highest professional qualification. Using the CASMIN educational classification scheme [22], we differentiated between three levels of education: low (CASMIN 1: primary and low secondary education), medium (CASMIN 2: intermediate/high secondary education), and high (CASMIN 3: tertiary education).

### Statistical methods

The prevalence of sporting inactivity was estimated for each survey year, and stratified by sex and education. The European Standard Population (ESP) according to Eurostat [23] was used to calculate sex- and age-standardised prevalence rates to account for demographic changes over time and for differences in the sex and age distribution between the education groups. Accordingly, the standardised prevalence rates reported in this article represent the percentage of people without sports participation in different groups, on the assumption that each group has the same age (and sex) structure. The standardised prevalence rates therefore enable comparisons to be made across groups and time, taking into account the differences in the age (and sex) structure of the groups compared. To test for the statistical significance of a trend over time from 2003 to 2012, we computed *p*-values for the linear effect of the survey year on sporting inactivity, as estimated by logistic regression.

Trends in educational inequalities in sporting inactivity were analysed using simple measures of group differences (prevalence difference [PD], odds ratio [OR]) as well as complex summary measures of inequality (Slope Index of Inequality [SII], Relative Index of Inequality [RII]). While PDs and SIIs quantify the magnitude of absolute inequality, ORs and RIIs represent the magnitude of relative inequalities. Selective use of exclusively absolute or relative measures of inequality can lead to a biased assessment of increasing or decreasing health inequalities over time, which is why it is recommended to consider both whenever possible [16, 17].

To take account of the different age structures of the groups compared, the PDs were calculated based on the standardised prevalence rates (see above) and the ORs were adjusted for age by means of logistic regression analysis. Accordingly, the PDs reported in this article are the absolute differences of the standardised prevalence rates of sporting inactivity (in percentage points) between the education groups, while the ORs represent the ratios of the age-adjusted odds of sporting inactivity between the education groups.

The SII and RII are regression-based measures that take into account the entire distribution of a socioeconomic variable and the size of the socioeconomic groups [16, 24]. These features are particularly useful in monitoring health inequalities, as they make the measures sensitive to changes over time in the socioeconomic distribution of a population. We used generalised linear regression models for binomial data with an identity link function (logarithmic link function) to compute the SII (RII). The ordered categorical education variable was converted to a metric fractional rank variable ranging from 0 (most education) to 1 (least education) before including it as an independent variable in the regression models [12]. The resulting SII (RII) can be interpreted as the estimated prevalence difference (prevalence ratio) between people with the lowest and highest educational level. Time trends in the SII and RII were analysed by adding an interaction term between education and year to the models while adjusting for sex, age, sex × age, sex × year, age × year, and education. To test whether trends in inequalities differ between age groups (25–49 vs. 50–65[reference]) and according to sex, the following interaction terms were added to the models: education × year × age_group and education × year × age_group × sex.

We used weighting factors to account for unequal sampling probabilities and to adjust the distribution of each sample by sex, age, education, and region to match the official population statistics for Germany. Analyses were performed using the Stata 14.1 (StataCorp LP, College Station, TX) survey data commands. Results were considered statistically significant when *p* < 0.05.

## Results

Table 1 describes the characteristics of the study population by survey year. From 2003 to 2012, the mean age and educational attainment of the study population increased, while the sex ratio remained constant over time (Table 1). The crude prevalence of sporting inactivity declined from 39.4% in 2003 over 35.1% in 2009 and 35.9% in 2010 to 32.7% in 2012 (*p*-trend < 0.001). As can be seen in Table 2, this decline was found in both men and women. When stratified by education, the decline in the crude sporting inactivity prevalence was observed only in the high and medium education groups, while there was no significant trend in men and women with low education.

**Table 2:** Crude prevalence of sporting inactivity among adults in Germany aged 25–69 years by survey year

	2003	2009	2010	2012	*p*-trend
	% (95% CI)	% (95% CI)	% (95% CI)	% (95% CI)	
Total:					
Low education	47.9 (45.3–50.4)	46.5 (44.5–48.5)	48.6 (46.6–50.6)	48.4 (45.8–51.1)	0.839
Medium education	37.5 (35.5–39.5)	32.4 (31.1–33.8)	33.2 (31.9–34.5)	30.2 (28.9–31.6)	<0.001
High education	25.1 (22.9–27.5)	20.2 (18.9–21.5)	20.0 (18.8–21.3)	16.6 (15.3–17.9)	<0.001
Total	39.4 (38.0–40.8)	35.1 (34.1–36.1)	35.9 (34.9–36.8)	32.7 (31.6–33.8)	<0.001
Men:					
Low education	48.7 (45.1–52.4)	47.9 (45.0–50.8)	49.6 (46.7–52.5)	49.8 (46.1–53.4)	0.740
Medium education	38.9 (35.8–42.1)	33.8 (31.7–36.0)	35.5 (33.4–37.7)	32.3 (30.2–34.4)	0.001
High education	24.7 (21.8–27.8)	20.4 (18.6–22.4)	19.8 (18.1–21.5)	15.8 (14.2–17.5)	<0.001
Total	39.9 (37.9–42.0)	36.1 (34.6–37.7)	37.1 (35.6–38.5)	33.9 (32.4–35.6)	<0.001
Women:					
Low education	47.0 (43.4–50.6)	45.0 (42.2–47.7)	47.5 (44.8–50.2)	46.9 (43.0–50.8)	0.930
Medium education	36.3 (33.8–38.8)	31.3 (29.7–32.9)	31.2 (29.7–32.8)	28.4 (26.6–30.3)	<0.001
High education	25.8 (22.3–29.7)	19.9 (18.2–21.7)	20.4 (18.7–22.2)	17.5 (15.5–19.6)	<0.001
Total	38.8 (36.9–40.8)	34.0 (32.8–35.3)	34.7 (33.4–35.9)	31.5 (29.9–33.1)	<0.001

The age- and sex-standardised prevalence rates shown in Table 3 confirmed the significant decline in sporting inactivity over the study period. Overall, and for men and women separately, the standardised prevalence of inactivity declined significantly between 2003 and 2012. In each survey year, the results show a strong social gradient: the lower the educational level of men and women, the higher the standardised sporting inactivity prevalence. While the standardised prevalence declined in the high and medium education groups, it remained constant in the low education group. This pattern was found in both men and women.

**Table 3:** Standardised prevalence of sporting inactivity among adults in Germany aged 25–69 years by survey year

	2003	2009	2010	2012	*p*-trend
	%^a^ (95% CI)	%^a^ (95% CI)	%^a^ (95% CI)	%^a^ (95% CI)	
Total:					
Low education	48.1 (45.4–50.9)	46.9 (44.8–49.1)	48.2 (46.1–50.3)	48.7 (45.7–51.6)	0.865
Medium education	37.5 (35.4–39.6)	32.9 (31.5–34.2)	33.5 (32.2–34.9)	30.9 (29.4–32.3)	<0.001
High education	25.0 (22.6–27.6)	20.1 (18.8–21.4)	19.9 (18.7–21.1)	16.4 (15.2–17.7)	<0.001
Total	39.4 (38.0–40.8)	35.0 (34.0–36.0)	35.7 (34.8–36.7)	32.7 (31.6–33.8)	<0.001
Men:					
Low education	47.8 (43.9–51.7)	46.7 (43.6–49.8)	48.4 (45.4–51.5)	49.5 (45.6–53.4)	0.584
Medium education	40.4 (37.1–43.9)	35.4 (33.2–37.8)	37.1 (34.8–39.4)	33.7 (31.6–36.0)	0.002
High education	24.8 (21.8–28.2)	20.3 (18.5–22.3)	19.5 (17.8–21.2)	15.9 (14.3–17.7)	<0.001
Total	40.1 (38.0–42.2)	36.3 (34.8–37.8)	37.0 (35.5–38.5)	34.1 (32.5–35.8)	<0.001
Women:					
Low education	48.5 (44.5–52.5)	47.1 (44.1–50.1)	48.0 (45.0–50.9)	47.8 (43.3–52.3)	0.783
Medium education	35.6 (33.0–38.2)	31.4 (29.8–33.0)	31.5 (29.9–33.1)	28.5 (26.7–30.4)	<0.001
High education	25.3 (21.4–29.6)	19.8 (18.1–21.7)	20.3 (18.6–22.0)	17.1 (15.2–19.1)	<0.001
Total	38.8 (36.9–40.8)	34.0 (32.8–35.3)	34.7 (33.5–36.0)	31.3 (29.7–32.9)	<0.001

^a^Standardised to the European Standard Population 2013, by age (and sex) [see also the Statistical methods subsection of the Methods section]

Table 4 presents the measures of absolute educational inequalities in sporting inactivity. The standardised PD between the low and high education group increased from 23.1 percentage points in 2003 to 32.2 in 2012. The PD between the medium and high education groups remained relatively constant at a level between 12.5–14.4 percentage points. The overall SII for sporting inactivity increased gradually from 0.30 in 2003 to 0.41 in 2012. Adding an interaction with age group (term: education × year × age_group) to the model showed that the increase in absolute inequalities was larger in the younger under-50 age group than in the age group of 50 and above (coefficient = 0.05; *p* = 0.013). In the sex-specific analysis, significant increases over time in the PDs between the high and low education group were observed in both men and women. The increase in the SII was significant only in men, while in women the SII increase was not statistically significant at the 5% level. The interaction of the trend with age group and sex (term: education × year × age_group × sex) was not statistically significant (*p* = 0.474), suggesting that the larger increase in absolute inequalities among the younger age group was similar in men and women.

**Table 4:** Absolute educational inequalities in sporting inactivity among adults in Germany aged 25–69 years by survey year

	2003	2009	2010	2012	*p*-trend
Total:					
PD^a^ (95% CI)					
Low education	23.1 (19.4–26.9)	26.9 (24.4–29.4)	28.3 (25.9–30.8)	32.2 (29.0–35.5)	<0.001
Medium education	12.5 (9.2–15.8)	12.8 (10.9–14.7)	13.7 (11.9–15.5)	14.4 (12.5–16.4)	0.225
High education (ref.)	0.0	0.0	0.0	0.0	
SII^b^ (95% CI)	0.30 (0.25–0.35)	0.34 (0.31–0.38)	0.38 (0.34–0.41)	0.41 (0.37–0.45)	0.001
Men:					
PD^a^ (95% CI)					
Low education	22.9 (17.9–28.0)	26.4 (22.8–30.1)	29.0 (25.4–32.5)	33.6 (29.3–37.9)	0.001
Medium education	15.6 (10.9–20.3)	15.1 (12.1–18.1)	17.6 (14.8–20.5)	17.8 (15.0–20.6)	0.299
High education (ref.)	0.0	0.0	0.0	0.0	
SII^c^ (95% CI)	0.30 (0.23–0.38)	0.36 (0.30–0.41)	0.39 (0.34–0.44)	0.44 (0.39–0.49)	0.004
Women:					
PD^a^ (95% CI)					
Low education	23.2 (17.5–28.9)	27.3 (23.8–30.8)	27.7 (24.3–31.1)	30.7 (25.8–35.6)	0.041
Medium education	10.3 (5.4–15.1)	11.6 (9.1–14.0)	11.2 (8.9–13.6)	11.4 (8.7–14.2)	0.637
High education (ref.)	0.0	0.0	0.0	0.0	
SII^c^ (95% CI)	0.29 (0.22–0.37)	0.33 (0.29–0.38)	0.36 (0.32–0.41)	0.37 (0.32–0.43)	0.077

*PD* prevalence difference (in percentage points), *SII* Slope Index of Inequality; ref., reference group

^a^standardised to the European Standard Population 2013, by age (and sex) [see also the Statistical methods subsection of the Methods section]

^b^Adjusted for age, sex, age × sex

^c^Adjusted for age

The measures of relative educational inequalities in sporting inactivity are shown in Table 5. In the total population, the age- and sex-adjusted OR of sporting inactivity in the low vs. high education groups increased from 2.80 to 4.63 between 2003 and 2012. The OR in the medium vs. high education groups showed an increase from 1.85 to 2.28. The overall RII for sporting inactivity was 2.08 in 2003 and increased gradually to 3.44 in 2012. Adding an interaction with age group (education × year × age_group) to the model revealed that the increase in relative inequalities was larger in the under-50 age group than among the elderly aged 50 and above (exp(b) = 1.26; *p* < 0.000). When the analysis was stratified by sex, similar results were found for men and women; the OR in the low vs. high education group and the RII increased significantly in both sexes. The interaction of the trend in inequalities with age group and sex (education × year × age_group × sex) was not statistically significant (*p* = 0.280), so that the larger increase in relative inequalities among the younger age group was found not to differ between men and women.

**Table 5:** Relative educational inequalities in sporting inactivity among adults in Germany aged 25–69 years by survey year

	2003	2009	2010	2012	*p*-trend
Total:					
OR^a^ (95% CI)					
Low education	2.80 (2.38–3.30)	3.47 (3.08–3.90)	3.78 (3.37–4.23)	4.63 (4.01–5.36)	<0.001
Medium education	1.85 (1.59–2.16)	2.01 (1.81–2.23)	2.09 (1.89–2.30)	2.28 (2.03–2.56)	0.046
High education (ref.)	1.00	1.00	1.00	1.00	
RII^a^ (95% CI)	2.08 (1.83–2.38)	2.58 (2.32–2.87)	2.79 (2.53–3.07)	3.44 (3.03–3.91)	<0.001
Men:					
OR^b^ (95% CI)					
Low education	2.80 (2.26–3.48)	3.47 (2.93–4.10)	3.88 (3.31–4.54)	5.10 (4.20–6.19)	<0.001
Medium education	2.06 (1.66–2.54)	2.17 (1.86–2.53)	2.40 (2.08–2.78)	2.73 (2.33–3.20)	0.070
High education (ref.)	1.00	1.00	1.00	1.00	
RII^b^ (95% CI)	2.06 (1.72–2.47)	2.50 (2.16–2.90)	2.73 (2.38– 3.13)	3.45 (2.92–4.07)	<0.001
Women:					
OR^b^ (95% CI)					
Low education	2.75 (2.14–3.53)	3.44 (2.93–4.04)	3.60 (3.08–4.21)	4.11 (3.30–5.11)	0.017
Medium education	1.64 (1.31–2.04)	1.85 (1.62–2.11)	1.78 (1.56–2.02)	1.87 (1.58–2.21)	0.335
High education (ref.)	1.00	1.00	1.00	1.00	
RII^b^ (95% CI)	2.13 (1.76–2.58)	2.71 (2.34–3.14)	2.89 (2.52–3.32)	3.46 (2.84–4.23)	0.001

*OR* odds ratio, *RII* Relative Index of Inequality; ref., reference group

^a^Adjusted for age, sex, age × sex

^b^Adjusted for age

## Discussion

To our knowledge, this is the first study to investigate whether and how the magnitude of absolute and relative educational inequalities in sporting inactivity has changed among adults in Germany since the early 2000s. The findings indicate that the overall prevalence of sporting inactivity declined between 2003 and 2012, but that this decline was solely due to a decrease in sporting inactivity among the better educated. In the low education group, the prevalence of sporting inactivity was consistently high throughout the study period. Hence, the sporting inactivity gap between adults with high and low educational attainment in Germany has widened significantly on both the absolute and relative scale within less than a decade. As our findings indicate, these trends were not sex-specific, but occurred similarly in both men and women. However, the increase in inequalities was larger in younger age groups under 50 than among the elderly population.

### Strengths and limitations

The analyses in this study were based on large nationwide samples, which enabled separate analysis for men and women. Owing to the sample design and the weighting factors used to adjust for survey non-response, it is possible to draw conclusions for the adult population aged 25 to 69 years in Germany from our results. That the methods of data collection did not change across surveys assured adequate comparability of data over time. Recall bias should have been low, as the questions on education referred to the present and the recall period for sporting inactivity was only 3 months. The use of internationally established methods, such as the CASMIN classification, the ESP 2013, and the SII and RII as summary measures for the magnitude of inequality, can enable other researchers to use our results for between-study and cross-country comparisons, or future meta-analyses.

There are several limitations worth noting in this study. First, causality between education and sporting inactivity cannot be inferred because of the observational nature of the data. Second, the intervals between the surveys were not equal, which may have potentially biased the results for time trends. Third, it must be considered that the data on sporting inactivity were based on self-reports. Self-report measures of physical activity have only a low-to-moderate correlation with direct/objective activity measures [25], which may be due to the relatively large amount of cognitive effort required to answer questions on physical activity [26]. This, however, applies primarily to detailed questions on frequencies and durations of different activity types, whereas a simple yes/no question about any sports activity during the last 3 months should be less affected as it is less cognitively demanding. Furthermore, self-reports on sports activity can be subject to social desirability bias resulting in a potential under-reporting of sporting inactivity [27, 28]. If better-educated participants were more likely to under-report their inactivity than less-educated participants, our results could potentially overestimate the extent of educational inequalities in sporting inactivity. If the perceived social unacceptability of sporting inactivity increased disproportionately in more highly-educated groups during the study period, a resulting increase in socially desirable responses from better-educated participants may have contributed to the observed widening of inequalities in sporting inactivity.

It must further be taken into account that the binary variable of sporting inactivity during the last 3 months (yes vs. no) used as the outcome measure in our study is a rather crude indicator for lack of health-enhancing physical activity. The results therefore do not allow conclusions to be drawn either on how inequalities in total sports activity levels have changed, or on trends in inequalities in the frequency and duration of activity. However, we performed a sensitivity analysis using a binary variable of up to 2 h vs. more than 2 h of sports activity per week as an outcome measure. Although the sensitivity analysis generally shows smaller inequalities in sports (in)activity, they are in line with the results from the main analysis in indicating significant increases in inequalities on both the absolute and relative scale for men and women. Another trend study in which the binary yes/no indicator of sporting inactivity during the last 3 months was used indicated that sporting inactivity has continuously decreased between the German Health Interview and Examination Surveys 1990–92, 1997–99, and 2008–11 [29]. This is consistent with our findings, which indicates good reliability of the results produced by this indicator. Evidence supporting the convergent validity of our findings (e.g. co-occurring increasing obesity inequalities according to education, as a result of increasing sports inequalities) is, to our best knowledge, not available to date for the German adult population.

Moreover, it has to be acknowledged that the term “sport” was not precisely defined in the interview and/or no word list was provided. As a consequence, the definition of the term was up to each respondent’s subjective concept of sport. However, as mentioned in the Methods section, in German the term “sport” is a very broad term; it is generally thought to include not only competitive club sports but also physical exercise to improve or maintain one’s physical fitness. From our knowledge, this is a wider understanding than is common in the English language, which must be borne in mind with regard to the results of our study.

Concerning the external validity and national representativeness of the samples, it must be mentioned that the response rate decreased across the surveys. However, the sample bias according to sex, age, education, and region, increased only slightly between 2003 and 2009 and remained constant thereafter, as indicated by the overall sample efficiency [see Additional file 1: Table S1]. To minimise the impact of potential selection bias from differential non-response across the surveys, we adjusted for non-response year-specifically by using weighting factors (see above). As the weighting procedure takes into account the age, sex, educational, and regional distribution of the samples, their national representativeness is limited to these characteristics.

### Comparison with other research

The findings of this nationwide study are consistent with previous studies indicating that sporting inactivity and other types of physical inactivity have decreased in many developed countries over the last decades [30–34]. However, the picture with regard to changes in social inequalities in inactivity habits is less coherent according to previous research from developed countries. While some studies have found indications of widening inequalities consistent with ours [30, 32, 35], others have found inequalities during the last 2–3 decades to be constant [31, 33, 36, 37]. A trend study from Canada examined absolute and relative inequalities in leisure-time physical inactivity and suggested that both kinds of inequalities have narrowed from 1981 to 1996, but widened from 1996 to 2005 [38]. A study conducted in a US metropolitan population suggests a narrowing of inequalities during the 1980s [39]. Scholes and colleagues [40] have examined trends in area-level socioeconomic inequalities in physical activity levels (without work-based activity) in England from 1998 to 2008. In contrast to our findings, they found unchanged absolute and relative inequalities among younger groups and a widening of absolute inequalities among older women. The partial inconsistency in findings may be due to between-study differences in inactivity measures, study periods, countries/regions, and indicators of social position. Moreover, most studies have considered only either absolute or relative inequalities, which can have a significant impact on judgements about inequality trends [17]. Mixed results on trends in inequalities in leisure-time physical inactivity, however, have also been found between different US states within the same study [35]. Altogether, this suggests that trends in social inequalities in physical inactivity habits can be subject to strong contextual, period, cohort, sociocultural, and/or regional differences. A regional study conducted in northeast German rural communities indicated that the educational gradient in adult sporting inactivity evolved in this part of the former East Germany only in the course of social transformation after German reunification [41]. Data from a retrospective survey of elderly residents in two German cities, similar to our nationwide findings, indicate a stronger increase in sports participation in the better-educated, particularly since the end of the 1990s [34].

Our observation of widening educational inequalities in sporting inactivity can be regarded as another example of “diffusion of innovations” [42]. According to this theory, persons of higher social position tend to adopt modern ideas (e.g. a health-conscious and physically active lifestyle) earlier or more quickly than those of a lower social position because, among other reasons, they can draw on more resources. For example, members of advantaged social groups may be able to adopt more healthy behaviours at a faster pace because they are more likely to live in supportive environments that place fewer constraints on individual choice than those faced by people in disadvantaged groups [43]. Along with this idea from diffusion theory, the theory of fundamental causes [44, 45] may help explain why social inequalities in sporting inactivity persist over time despite progressive public initiatives to promote physically active lifestyles in the population. Fundamental cause theory argues that the association between social position and health is time-persistent because the lack of material and non-material resources (e.g. money and knowledge) in lower social groups, which is regarded as the fundamental cause of the association, persists no matter how other factors and circumstances may change (e.g. public initiatives to promote physical activity, sports promotion strategies, or physical activity promoting environments). In addition to different resources for pursuing the goal of good health, the theory considers different preferences for health as one of the “metamechanisms” that may contribute to the durable association between social position and health [46]. People in higher social groups may exhibit a stronger and more consistent preference for future good health than people in lower social groups, for example, because of different time horizons or as part of their cultural “habitus” [46, 47]. Different preferences for health could also impact on the motivation to commence and maintain sports activity in adulthood and may explain why sporting inactivity declines faster or earlier in higher social groups.

Another explanation often given in the context of educational disparities in health behaviours is the unequal distribution of health-related knowledge. Smith et al. [38] argue, however, that widening education-related inequalities in physical activity habits are unlikely to reflect unequal knowledge of the consequences of unhealthy behaviours because the basic relevant knowledge is widespread in all education groups today. They emphasize, instead, that widening gaps in health behaviours between groups with different education levels highlight some of the challenges faced by public health interventions. Population-level interventions that seek to improve the health of a population can have unequal effects across social groups and thereby lead to unintended exacerbations of health inequalities [48]. With particular respect to population-based physical activity interventions, however, a systematic review shows that sufficient information on social group differences in their effectiveness is still lacking [49]. Therefore, existing and future population-based interventions to promote physical and sports activity should be accompanied by scientific monitoring to ascertain whether they are more effective in some social groups than in others. The resulting findings could help identify effective strategies to promote sports activity in socially disadvantaged groups and thereby to reduce social inequalities in inactivity habits.

Furthermore, it needs to be borne in mind that sporting activity, as a subset of leisure-time physical activity, is only a small component of total physical activity level. Disadvantaged social groups are often more physically active at work and in everyday life, and have thus a higher total energy expenditure level compared with privileged social groups [5]. This could also explain why they are less likely to participate in leisure sports activity and leisure physical activity interventions. However, participation in sports activities is still desirable because of the greater health benefits associated with these types of activity compared with work-related physical activity [1].

## Conclusions

In summary, this study provides evidence that the prevalence of sporting inactivity declined in the German adult population between 2003 and 2012, but that the educational gap in sporting inactivity persisted and even widened during this period. The widening of inequalities was larger in younger age groups than among the elderly adult population, indicating that this trend is particularly due to rising sports participation among higher-educated people from younger cohorts. Although the results of this study do not confirm a causal contribution by Germany’s physical activity initiative to the observed widening of inequalities in sports participation, it can be concluded that the initiative at least may not have narrowed the sporting gap between different education groups so far. Of course, from a public health perspective, the overall decline in sporting inactivity is a positive development. However, this decline was solely due to an increase in sports participation among only the better educated. As social inequalities in health-enhancing physical activity contribute to social inequalities in health outcomes [11], effective physical activity interventions that are specifically targeted to less-educated younger adults are required to prevent future increases in social inequalities in health and disease.

## Additional file

Additional file 1: Key survey metrics. (PDF 225 kb)

## Abbreviations

CASMIN: Comparative Analysis of Social Mobility in Industrial Nations
ESP: European Standard Population
GEDA: German Health Update study
OR: Odds ratio
PD: Prevalence difference
RII: Relative Index of Inequality
RKI: Robert Koch Institute
SII: Slope Index of Inequality

## References

[1] Andersen LB, Schnohr P, Schroll M, Hein HO (2000). All-cause mortality associated with physical activity during leisure time, work, sports, and cycling to work. Arch Intern Med.

[2] US Department of Health and Human Services (2008). 2008 physical activity guidelines for Americans: be active, healthy, and happy.

[3] World Health Organization (2011). Promoting sport and enhancing health in European Union countries: a policy content analysis to support action.

[4] Federal Ministry of Food Agriculture and Consumer Protection, Federal Ministry of Health (2013). IN FORM: German national initiative to promote healthy diets and physical activity.

[5] Finger JD, Tylleskär T, Lampert T, Mensink GBM (2012). Physical activity patterns and socioeconomic position: the German National Health Interview and examination survey 1998 (GNHIES98). BMC Public Health.

[6] Hoebel J, Finger JD, Kuntz B, Lampert T (2016). Sozioökonomische Unterschiede in der körperlich-sportlichen Aktivität von Erwerbstätigen im mittleren Lebensalter: Welche Rolle spielen Bildung, Beruf und Einkommen? [socioeconomic differences in physical activity in the middle-aged working population: the role of education, occupation, and income]. Bundesgesundheitsbl..

[7] Demarest S, Van Oyen H, Roskam A-J, Cox B, Regidor E, Mackenbach JP (2014). Educational inequalities in leisure-time physical activity in 15 European countries. Eur J Pub Health..

[8] Beenackers MA, Kamphuis CBM, Giskes K, Brug J, Kunst AE, Burdorf A (2012). Socioeconomic inequalities in occupational, leisure-time, and transport related physical activity among European adults: a systematic review. Int J Behav Nutr Phys Act..

[9] Gidlow C, Johnston LH, Crone D, Ellis N, James D (2006). A systematic review of the relationship between socio-economic position and physical activity. Health Educ J.

[10] Kamphuis CB, Van Lenthe FJ, Giskes K, Huisman M, Brug J, Mackenbach JP (2008). Socioeconomic status, environmental and individual factors, and sports participation. Med Sci Sports Exerc.

[11] Laaksonen M, Talala K, Martelin T, Rahkonen O, Roos E, Helakorpi S (2008). Health behaviours as explanations for educational level differences in cardiovascular and all-cause mortality: a follow-up of 60 000 men and women over 23 years. Eur J Pub Health.

[12] Harper S, Lynch J, Oakes JM, Kaufman JS (2006). Measuring health inequalities. Methods in social epidemiology.

[13] Mackenbach JP, Martikainen P, Menvielle G, de Gelder R (2016). The arithmetic of reducing relative and absolute inequalities in health: a theoretical analysis illustrated with European mortality data. J Epidemiol Community Health.

[14] Mackenbach JP (2015). Should we aim to reduce relative or absolute inequalities in mortality?. Eur J Pub Health.

[15] Mackenbach JP, Kulhánová I, Menvielle G, Bopp M, Borrell C, Costa G (2015). Trends in inequalities in premature mortality: a study of 3.2 million deaths in 13 European countries. J Epidemiol Community Health.

[16] Wagstaff A, Paci P, van Doorslaer E (1991). On the measurement of inequalities in health. Soc Sci Med.

[17] Harper S, King NB, Young ME (2013). Impact of selective evidence presentation on judgments of health inequality trends: an experimental study. PLoS One.

[18] Krug S, Jordan S, Mensink G, Müters S, Finger JD, Lampert T (2013). Physical activity: results of the German health interview and examination survey for adults (DEGS1). Bundesgesundheitsbl..

[19] Kurth B-M, Lange C, Kamtsiuris P, Hölling H (2009). Gesundheitsmonitoring am Robert Koch-Institut: Sachstand und Perspektiven [health monitoring at the Robert Koch Institute: status and perspectives]. Bundesgesundheitsbl..

[20] Lange C, Jentsch F, Allen J, Hoebel J, Kratz AL, von der Lippe E (2015). Data resource profile: German health update (GEDA)—the health interview survey for adults in Germany. Int J Epidemiol.

[21] Kohler M, Rieck A, Borch S (2005). Methode und design des telefonischen Gesundheitssurveys 2003 [method and design of the telephone health survey 2003]. Bundesgesundheitsbl.

[22] Brauns H, Scherer S, Steinmann S, Hoffmeyer-Zlotnik JHP, Wolf C (2003). The CASMIN educational classification in international comparative research. Advances in cross-national comparison: an European working book for demographic and socio-economic variables.

[23] Eurostat (2013). Revision of the European Standard Population: Report of Eurostat's task force (2013 edition).

[24] Mackenbach JP, Kunst AE (1997). Measuring the magnitude of socio-economic inequalities in health: an overview of available measures illustrated with two examples from Europe. Soc Sci Med.

[25] Prince SA, Adamo KB, Hamel ME, Hardt J, Gorber SC, Tremblay M (2008). A comparison of direct versus self-report measures for assessing physical activity in adults: a systematic review. Int J Behav Nutr Phys Act.

[26] Finger JD, Gisle L, Mimilidis H, Santos-Hoevener C, Kruusmaa EK, Matsi A (2015). How well do physical activity questions perform? A European cognitive testing study. Arch Public Health.

[27] Christensen AI, Ekholm O, Glümer C, Juel K (2014). Effect of survey mode on response patterns: comparison of face-to-face and self-administered modes in health surveys. Eur J Pub Health.

[28] Hoebel J, von der Lippe E, Lange C, Ziese T (2014). Mode differences in a mixed-mode health interview survey among adults. Arch Public Health.

[29] Finger JD, Busch MA, Du Y, Heidemann C, Knopf H, Kuhnert R (2016). Time trends in cardiometabolic risk factors in adults. Dtsch Arztebl Int.

[30] Hotchkiss JW, Davies C, Gray L, Bromley C, Capewell S, Leyland AH (2011). Trends in adult cardiovascular disease risk factors and their socio-economic patterning in the Scottish population 1995–2008: cross-sectional surveys. BMJ Open.

[31] Petersen CB, Thygesen LC, Helge JW, Gronbaek M, Tolstrup JS (2010). Time trends in physical activity in leisure time in the Danish population from 1987 to 2005. Scand J Public Health..

[32] Najman JM, Toloo G, Siskind V (2006). Socioeconomic disadvantage and changes in health risk behaviours in Australia: 1989-90 to 2001. Bull World Health Organ.

[33] Ding D, Do A, Schmidt H-M, Bauman AE (2015). A widening gap? Changes in multiple lifestyle risk behaviours by socioeconomic status in new South Wales, Australia, 2002–2012. PLoS One.

[34] Klostermann C, Nagel S (2014). Changes in German sport participation: historical trends in individual sports. Int Rev Sociol Sport.

[35] Harper S, Lynch J (2007). Trends in socioeconomic inequalities in adult health behaviors among U.S. states, 1990-2004. Public Health Rep.

[36] Galobardes B, Costanza MC, Bernstein MS, Delhumeau C, Morabia A (2003). Trends in risk factors for lifestyle-related diseases by socioeconomic position in Geneva, Switzerland, 1993-2000: health inequalities persist. Am J Public Health.

[37] Groth MV, Sørensen MR, Matthiessen J, Fagt S, Landvad N, Knudsen VK (2014). Disparities in dietary habits and physical activity in Denmark and trends from 1995 to 2008. Scand J Public Health.

[38] Smith P, Frank J, Mustard C (2009). Trends in educational inequalities in smoking and physical activity in Canada: 1974-2005. J Epidemiol Community Health.

[39] Iribarren C, Luepker RV, McGovern PG, Arnett DK, Blackburn H (1997). Twelve-year trends in cardiovascular disease risk factors in the Minnesota heart survey. Are socioeconomic differences widening?. Arch Intern Med.

[40] Scholes S, Bajekal M, Love H, Hawkins N, Raine R, O'Flaherty M (2012). Persistent socioeconomic inequalities in cardiovascular risk factors in England over 1994–2008: a time-trend analysis of repeated cross-sectional data. BMC Public Health..

[41] Röding D, Elkeles T (2016). Sportpartizipation in nordostdeutschen Landgemeinden in den Jahren 1973, 1994 und 2008 [sport participation in northeast German rural communities in 1973, 1994 and 2008]. Sportwissenschaft.

[42] Rogers EM (1983). Diffusion of innovations.

[43] Institute of Medicine (2003). The future of the public’s health in the 21st century.

[44] Link BG, Phelan J. Social conditions as fundamental causes of disease. J Health Soc Behav. 1995; special issue:80-94. 7560851

[45] Phelan JC, Link BG, Tehranifar P (2010). Social conditions as fundamental causes of health inequalities: theory, evidence, and policy implications. J Health Soc Behav.

[46] Freese J, Lutfey K, Pescosolido BA, Martin JK, McLeod JD, Rogers A (2011). Fundamental causality: challenges of an animating concept for medical sociology. Handbook of the sociology of health, illness, and healing: a blueprint for the 21st century.

[47] Mackenbach JP, Looman CWN, Artnik B, Bopp M, Deboosere P, Dibben C, et al. ‘Fundamental causes’ of inequalities in mortality: an empirical test of the theory in 20 European populations. Sociol Health Illn. 2017. 10.1111/1467-9566.12562 28369947

[48] Frohlich KL, Potvin L (2008). The inequality paradox: the population approach and vulnerable populations. Am J Public Health.

[49] Humphreys DK, Ogilvie D (2013). Synthesising evidence for equity impacts of population-based physical activity interventions: a pilot study. Int J Behav Nutr Phys Act.

