# Supplementary material for: Changing educational inequalities in sporting inactivity among adults in Germany: a trend study from 2003 to 2012
Source: BMC Public Health. 2017 Jun 6;17:547. doi: 10.1186/s12889-017-4478-2 (PMC5461755; doi:10.1186/s12889-017-4478-2)
Supplement: Additional file 1: — Key survey metrics. (PDF 225 kb) [file 12889_2017_4478_MOESM1_ESM.pdf]

**Additional file 1: Table S1.** Key survey metrics (response rates, sample efficiencies and deviations of the sample characteristics from the target population)

| | 2003 | | | 2009 | | | 2010 | | | 2012 | | |
|---|---|---|---|---|---|---|---|---|---|---|---|---|
| | **Sample** % | **Germany**[a] % | ***Diff*** | **Sample** % | **Germany**[a] % | ***Diff*** | **Sample** % | **Germany**[a] % | ***Diff*** | **Sample** % | **Germany**[a] % | ***Diff*** |
| Sex | | | | | | | | | | | | |
| Men | 46.8 | 50.3 | *-3.5* | 42.2 | 50.2 | *-8.0* | 42.8 | 50.2 | *-7.5* | 49.5 | 50.3 | *-0.8* |
| Women | 53.2 | 49.7 | *+3.5* | 57.8 | 49.8 | *+8.0* | 57.2 | 49.8 | *+7.5* | 50.5 | 49.7 | *+0.8* |
| Age group | | | | | | | | | | | | |
| 25–39 years | 32.5 | 35.4 | *-2.9* | 29.0 | 31.3 | *-2.3* | 29.3 | 30.7 | *-1.5* | 23.0 | 30.0 | *-7.0* |
| 40–49 years | 30.1 | 24.9 | *+5.2* | 30.0 | 27.6 | *+2.4* | 29.1 | 27.9 | *+1.2* | 26.7 | 27.5 | *-0.8* |
| 50–59 years | 19.8 | 19.4 | *+0.4* | 22.6 | 22.0 | *+0.6* | 22.9 | 22.6 | *+0.3* | 26.1 | 24.4 | *+1.7* |
| 60–69 years | 17.7 | 20.3 | *-2.6* | 18.5 | 19.1 | *-0.7* | 18.7 | 18.8 | *-0.1* | 24.2 | 18.2 | *+6.0* |
| Education | | | | | | | | | | | | |
| Low | 29.2 | 37.5 | *-8.3* | 21.9 | 33.5 | *-11.7* | 21.4 | 33.1 | *-11.8* | 17.5 | 28.6 | *-11.1* |
| Medium | 47.4 | 47.0 | *+0.4* | 49.5 | 50.1 | *-0.6* | 48.0 | 49.1 | *-1.1* | 49.9 | 51.3 | *-1.4* |
| High | 23.4 | 15.6 | *+7.8* | 28.7 | 16.4 | *+12.3* | 31.7 | 17.8 | *+12.9* | 32.6 | 20.1 | *+12.5* |
| Response rate[b] | – [c] | | | 29.1% | | | 28.9% | | | 22.1% | | |
| Sample efficiency[d] | 67.4% | | | 57.7% | | | 58.0% | | | 58.8%[e] | | |

*Diff* = Difference between sample and target population (Germany)

[a] extrapolated based on official data from the German Federal Statistical Office

[b] "Response Rate 3" of the American Association for Public Opinion Research (AAPOR): Standard Definitions - Final Dispositions of Case Codes and Outcome Rates for Surveys. AAPOR; 2008.

[c] cannot be retrospectively calculated according to the 2008 version of the AAPOR Standard Definitions; the response rate reported earlier for 2003 is therefore not comparable with those for 2009–2012

[d] the sample efficiency rate represents the sample bias according to sex, age, education and region (higher efficiency means lower sample bias)

[e] the 2012 sample was regionally stratified, the rate represents the mean efficiency over sample strata
